# Good girls and boys: findings from a cross-sectional survey on adolescent rights, relationships, and sexuality in an urban informal settlement in India

**DOI:** 10.1080/02673843.2017.1371613

**Published:** 2017-09-06

**Authors:** Nayreen Daruwalla, Tanvi Mishra, Neeta Karandikar, Shanti Pantvaidya, David Osrin

**Affiliations:** a Society for Nutrition, Education and Health Action, Mumbai, India; b Institute for Global Health, University College London Institute of Child Health, London, UK

**Keywords:** Adolescent, India, Mumbai, poverty areas, sexuality

## Abstract

Around 20% of India’s population are adolescents aged 10–19 years. Our objective was to strengthen program interventions on gender equity, health, and participation by gauging adolescents’ levels of understanding and opinions. In a cross-sectional survey, we interviewed 2005 adolescents on their opinions on rights, friendship and sex, sexual refusal and coercion, and communication with family, using a two-stage probability proportional to size sample. Opinions on gender allocations were generally equitable, although females supported clothing proscriptions. Premarital sex, multiple partners, masturbation and non-heterosexual partnerships were frowned upon. Few respondents said that they felt pressure to be sexually active, 79% said that sexual coercion was a form of violence, but 14% of older adolescents said that it would be unreasonable to refuse sex. Our interviews described young people negotiating the terrain between perceived normative expectations and contemporary aspirations, showing limited manoeuvring within assumed gender roles in which family control was prominent.

## Background

Of over 1.8 billion young people - adolescents (10–19 years) and youth (15–24 years) - in the world today, 90% live in developing countries, in 17 of which half the population is under 18 (UNFPA, 2014). Young people have tended to be underrepresented on the public agenda and have only recently been recognized as a target group of importance for population health. Adolescents make up about one-fifth of India’s population and young people about one-third, or 350 million (UNFPA, 2014).

Historically, the focus of government and non-government programs for young people in India has been on sexual and reproductive health. Programs have promoted awareness of sexual health and hygiene, communicable diseases such as sexually transmitted infections and HIV, nutrition, and contraception. The National Rural Health Mission (NRHM) includes adolescent sexual and reproductive health through interventions that emphasize group education strategies for family planning and maternal health. The Rashtriya Kishor Swasthya Karyakram (2014) includes nutrition, reproductive health, and substance abuse components (National Health Mission, 2014). The Adolescent Reproductive and Sexual Health (ARSH) initiative aims to reduce maternal and infant mortality rates, reduce the incidence of sexually transmitted infections (STIs), increase access to contraception, and delay the age of marriage, predominantly through knowledge and awareness as stimuli to improved care-seeking (National Rural Health Mission, 2006). Evaluation suggests that its success has been limited and that it has not succeeded in either increasing services for adolescents or improving the quality of the services they receive (Population Council & UniCEF, 2013). The recent National Programme for Youth and Adolescent Development (NPYAD) merges four schemes under the Ministry of Youth Affairs and Sports: Promotion of Youth Activities and Training, Promotion of National Integration, Promotion of Adventure and Development, and Empowerment of Adolescents (Government of India, 2015).

None of these initiatives has taken a particularly nuanced approach to gender and sexuality. Program content and approach tend not to address the structural and power inequities that women and girls face, and omit the development of skills to challenge socially accepted roles and expectations around sexuality, fertility, and work (Nanda, Das, Singh, & Negi, 2013). Sexuality is not prominent on their agendas and they have tended to reinforce hetero-normative behaviour and livelihoods, with the assumption of marriage as a young woman’s ultimate goal and little scope for informed choices about sexuality and personal space. Programs that have included young men have been fewer and have likewise posed few challenges to sexual and gender norms. Studies have shown that the parameters of hetero-normativity and social sanction for boys to engage in sexual behaviours are often related to conceptions of masculinity that manifest in authority and power (Abraham, 2001; Santhya, Haberland, Ram, Sinha, & Mohanty, 2007). The subtle aspects of their sexuality are often not explored. A study of constructs of masculinity and their influence on men’s wellbeing suggested that dominant norms and traditional beliefs about manhood were associated with greater risk-taking and risky sexual activity (Pradhan & Ram, 2010).

Despite restrictive social norms, there is increasing evidence that young people in India engage in premarital romantic and sexual partnerships (Jaya & HIndin, 2009). There are opportunities for social mixing, and young men and women have devised ways of developing romantic relationships, notwithstanding a social environment that ostensibly disapproves of interaction (Alexander, Garda, Kanade, Jejeebhoy, & Ganatra, 2007). TARSHI (Talking about Reproductive and Sexual Health Issues), a non-government organization running a helpline for sexual information, received over 59,000 calls from men seeking information on sexual anatomy and physiology in 2008. 33% of callers were young people (Tripathi & Sekher, 2013).

Home is a key place for sex education and parents’ attitudes are vital. Sex education in schools remains controversial. In 2007, the media reported that the Ministry of Health was considering a ban on sex education in schools since it conflicted with Indian cultural values and might lead adolescents to experimentation and irresponsible behaviour (McManus & Dhar, 2008; Tripathi & Sekher, 2013). As recently as 2014, newspapers carried the story that the government of Maharashtra had yet to implement proposals for sex education and that public schools had no structure within which to deliver it (Porecha, 2014). People tend to think of sex education as confined to information on anatomical and biological differences, rather than gender and sexuality. A WHO report (2003) suggested that promotion of family life and sex education had resulted in delay in age of entry into sexual relationships, reduced partner numbers, and increased safe sex and contraception. Sex education in schools did not seem to encourage younger people to have sex. Nevertheless, both proponents and critics of sex education in Indian schools use the language of ‘sexual restraint’, delaying the initiation of sexual activity until marriage (Tripathi & Sekher, 2013).

Information on how young people might begin and nurture relationships is scarce, though vital to address their needs. In the absence of other sources, young people rely on films and same-sex peers for information (Jaya & HIndin, 2009; Nair, Leena, George, Thankachi, & Russell, 2013b). As a result, they are likely to be poorly informed or misinformed. Despite evidence that appropriate information delays sexual initiation, prevents unwanted pregnancies, and lowers rates of STI transmission, social norms discourage discussion of issues related to sexuality in family settings, as parents often believe that this would imply approval. The cycle of poor communication keeps young people ill-informed and unlikely to receive parental support in sexual matters (Jaya & HIndin, 2009).

Research with adolescents has focused on access to health services, their knowledge of reproductive and sexual health, premarital partnerships, and risky sexual behaviours. Sexual health and wellbeing can be enhanced when adolescents are encouraged to express their sexuality candidly, and if they have been provided guidance on dealing with the issues positively. There is a need to create a social environment that allows expression of sexuality as a natural and important part of growing up. Such an environment will put adolescents in a better position to negotiate control and power over their sexuality, and will foster intolerance of violence.

A non-government organisation based in Mumbai, Society for Nutrition, Education and Health Action (SNEHA) manages the Empowerment, Health, and Sexuality for Adolescents (EHSAS) initiative with adolescents aged 11–19 years. The initiative includes individual and group activities and classroom sessions aimed at education on self-awareness, sexuality and its expression, soft and social skills, employability, and creative expression of sexuality through art and recreational activities. Group work with parents is conducted to create an enabling environment in which their maturing children can express their sexuality and build acceptance of it. Prior to the project, we did a needs-assessment to understand adolescents’ knowledge and practices around sexuality, gender and violence. Our objective was to provide background information for our work in project EHSAS. We were beginning the intervention program on adolescent health and sexuality and wanted to gauge levels of understanding and opinions in order to develop learning materials and intervention strategies.

## Methods

### Setting

The study was done in Dharavi, an urban informal settlement in Mumbai with an estimated population of 750,000. Informal settlements (slums) are features of urbanization in India and have been described in two-thirds of cities and towns. The most recent estimate is that 41% of Mumbai’s households are in such settlements (Chandramouli, 2011). UN-HABITAT characterizes them in terms of overcrowding, insubstantial housing, insufficient water and sanitation, lack of tenure, and hazardous location (Ministry of Housing and Urban Poverty Alleviation, 2010; United Nations Human Settlements Programme (UN-Habitat), 2003).

Dharavi covers 557 acres and is divided into 96 geographical clusters (Lewis, 2011). Our work with adolescents began on the backdrop of an existing program on prevention of violence against women and children, which itself began in 2000. Secondary interventions are provided through five counselling centres across Mumbai, linked with community mobilisation, health service, police, and legal support. Primary prevention is carried out through community mobilisation, mainly through group work and voluntarism. We currently run 180 groups of women, adolescents, and men. Key actions of these groups include collective support by community members and referral of survivors to support services.

Around 50 adolescent groups have been campaigning in communities since 2002. They carry out safety audits, mount performances and campaigns, undertake peer counselling, and participate in gender transformation programmes. More than a decade of work in the community means that our organisation is relatively visible in terms of our intent and credibility.

### Design

We did a cross-sectional quantitative survey of adolescents’ awareness of and opinions on rights, friendship and sex, sexual refusal and coercion, communication with parents, and general communication within families.

### Participants

The inclusion criteria were that respondents be aged 11–19 years, irrespective of socioeconomic status, caste, religion, gender, disability, or education. Unfortunately, we were unable to interview adolescents with cognitive, hearing, or speech impairments because the interview team did not have the necessary skills to do so.

### Tools

Questionnaire content was developed after focus group discussions with parents and adolescents divided into groups aged 11–14 and 15–19 years. The discussions addressed perceptions and practices around sexuality and sexual behavior, and the influence of gender, culture, and economic status on sexuality. Four areas of enquiry emerged: current awareness of rights, negotiation of control in relationships, levels of understanding of sexuality and opinions on it, and communication between adolescents and their families. The resulting questionnaire included six groups of predominantly closed categorical responses: family background, knowledge of rights, knowledge of and attitude to gender and violence, response to the participant’s own and others’ feelings of sexuality, and family influence and control over decision-making.

### Procedures

We recruited 38 field interviewers after written tests and two rounds of interviews. They were evaluated on their writing and communication skills, non-verbal communication, and understanding of relevant issues, and given three days of training on issues around sexuality, sex and gender, counseling and initial response, and technical aspects of administering the questionnaire. We piloted 100 interviews in the presence of a supervisor who intervened if the interviewer appeared to be struggling. At the end of each day, the supervisor went through the questionnaires, identified problems in completion, and checked if the investigator had any doubts or concerns.

The subsequent survey was done by two teams, one for males and one for females, each allotted a supervisor who observed interviews throughout data collection and checked questionnaires for data quality. The team of interviewers began at a central point in each cluster and worked in pairs, visiting every 50 home on the left-hand side of a lane and visiting adjacent homes if no adolescent lived there. If this process did not yield sufficient potential interviewees, they made enquiries about adolescents in the lane. Interviews took between 45 min and one hour, and interviewers arranged to come back if they were incomplete. Given the sensitive nature of the interview, we assumed the possibility of social desirability bias. We arranged that interviewers be unknown to respondents. The questionnaire was administered in respondents’ homes after signed consent from both them and their parents. We tried to conduct interviews without other people present. If a location such as a loft or verandah was not available, we booked a small hall in the vicinity of the cluster to afford respondents privacy. Interviewers were trained to avoid cues in body language or tone of voice that might influence respondents’ answers. Interviews were reviewed and compared by supervisors and field research coordinator at the end of each day, and flagged for interviewer review if there were discrepancies. The two groups met every two weeks to compare data collection and recording procedures.

### Sample size and selection

A sample size of 1000 in each of two groups (older compared with younger adolescents, or females compared with males) would have at least 90% power to detect a difference between groups of 8% in binary outcome proportions across the possible range of comparisons. We aimed, therefore, to achieve a two-stage sample of 2000 after interviewing 20–25 adolescents in each of 80 primary sampling units selected from 96 geographical clusters. The self-weighting sample of clusters was developed through Probability Proportional to Estimated Size (PPES), based on a population estimate of 750,000, an estimate that 20% of the population would be adolescent (the estimate from the third National Family Health Survey was 19% for households in slum areas of Maharashtra state; IIPS and Macro International, 2008), a sampling interval of 2981 and the first primary sampling unit defined by a random number between one and 2981.

### Data management and analysis

Survey questionnaires and information from interviews were kept in locked cupboards. Anonymized electronic data were entered into a database in Microsoft Access (Microsoft Corporation) and stored on a server in intelligibly named files. Information was backed up weekly on an external hard drive. Only the program director and senior members of the research team were allowed access, passwords were changed fortnightly, and data were read-only where possible. No datasets included the names of respondents. We tabulated frequencies and proportions of outcome variables, by sex and age (11–14 years and 15–19 years) and for the whole sample. We compared response proportions and 95% confidence intervals for differences between older and younger age groups and between females and males using survey commands in Stata 13 (College Station, TX), followed by lincom commands (linear combinations of estimators). We summarised socioeconomic position by quintiles of asset indices derived from standardized weights for the first component of a principal components analysis of household durables (Filmer & Pritchett, 2001; Vyas & Kumaranayake, 2006).

### Ethical concerns

Approval for the study was granted by the Multi-institutional Ethics Committee, Mumbai. The issue of sexuality required careful handling. The process of recalling distressing events might have disturbed interviewees, and there were family, social, and legal issues around the discussion. We were reassured when parents’ involvement in formative focus group discussions was enthusiastic, but we piloted the questionnaire extensively to gauge potential response. We had already worked with adolescents in the area and a support network of counselors, community peer workers, and healthcare professionals was in place for respondents to call upon. We trained the interviewers on the issues around confidentiality and ensured confidential data management. We discussed the study with community leaders before the focus group discussions, and subsequently organized meetings with community representatives in each cluster involved in the survey.

Informed consent was obtained from adolescents and their parents. Potential respondents were allowed a day to decide, along with their parents, whether they would like to be involved. Interviewers made sure that they had an opportunity to ask questions and understood the study. Respondents received no financial compensation. They were given information and booklets about HIV and child sexual abuse, and were provided with our counseling centre helpline numbers. If the respondent reported abuse, subsequent visits were made to the home to arrange a meeting with a counselor and appropriate follow-up.

## Results

We interviewed 2005 adolescents between 13 November 2013 and 8 June 2014. We had approached 3600 adolescents and 1600 declined to participate. Ten respondents were married: one male aged 19 and nine females aged 17–19. Table 1 summarises respondent characteristics. More of their mothers (28%) than fathers (13%) had not been to school. Substantial numbers of fathers were engaged in unskilled work (37%), work as machine operators, assemblers, or drivers (17%), or were skilled artisans (16%). Mothers tended to work in the home, including piecework (73%), but few were skilled artisans (<1%). There was a slight excess of males from families in higher socioeconomic quintiles. When asked about caste, 75% of respondents described theirs as either ‘open’ or ‘other.’ Most had been born in Mumbai (72%) and 85% said that they were at school. Of these, 58% (982) were at private schools and 8% (138) at municipal schools. More males (63%: 544) than females (50%: 420) were at English medium schools. Hindi (15%: 249) and Marathi (15%: 260) medium schools were represented equally, but more girls (14%: 122) than boys (8%: 67) were studying in Urdu. Less than 10% of adolescents said that they did paid work: 44% of these worked full-time, usually for less than Rs 200 per day. More males (39%) than females (16%) used the internet, most commonly through mobile phones (24%).

**Table 1.** Characteristics of adolescent respondents and their families.

	Male (%)	Female (%)	All (%)
Age group			
11–14	455 (47)	466 (45)	921 (100)
15–19	518 (53)	566 (55)	1084 (100)
Paternal education			
No schooling	103 (11)	149 (15)	252 (13)
Primary	226 (23)	229 (22)	455 (23)
Secondary	424 (43)	452 (44)	876 (44)
Higher	105 (11)	106 (10)	211 (10)
Other or unknown	115 (12)	96 (9)	211 (10)
Maternal education			
No schooling	249 (26)	315 (31)	564 (28)
Primary	290 (30)	271 (26)	561 (28)
Secondary	279 (29)	372 (36)	651 (33)
Higher	72 (7)	54 (5)	126 (6)
Other or unknown	83 (8)	20 (2)	103 (5)
Paternal livelihood			
Unemployed	30 (3)	37 (4)	67 (3)
Unskilled work	378 (39)	365 (35)	743 (37)
Plant or machine operator, assembler, driver	147 (15)	190 (18)	337 (17)
Skilled artisan	147 (15)	167 (16)	314 (16)
Service work	114 (12)	121 (12)	235 (12)
Other	73 (7)	48 (5)	121 (6)
Unknown	84 (9)	104 (10)	188 (9)
Maternal livelihood			
Unemployed	48 (5)	29 (3)	77 (4)
Unskilled work or homemaker	745 (77)	716 (69)	1461 (73)
Plant or machine operator, assembler, driver	83 (8)	206 (20)	289 (14)
Skilled artisan	2 (<1)	6 (<1)	8 (<1)
Service work	4 (<1)	2 (<1)	6 (<1)
Other	67 (7)	51 (5)	118 (6)
Unknown	24 (3)	22 (2)	46 (2)
Socioeconomic asset quintile			
Lowest	214 (22)	187 (18)	401 (20)
2	147 (15)	266 (26)	413 (21)
3	174 (18)	230 (22)	404 (20)
4	200 (21)	188 (18)	388 (19)
Highest	238 (24)	161 (16)	399 (20)
Religion			
Hindu	572 (59)	629 (61)	1201 (60)
Muslim	343 (35)	339 (33)	682 (34)
Christian	37 (4)	40 (4)	77 (4)
Buddhist	10 (1)	15 (1)	25 (1)
Other	11 (1)	9 (1)	20 (1)
Caste category			
Open	389 (40)	386 (37)	775 (39)
Scheduled caste	154 (16)	172 (17)	326 (16)
Other backward class	45 (5)	70 (7)	115 (6)
Scheduled tribe	9 (1)	23 (2)	32 (2)
Nomadic tribe	4 (<1)	20 (2)	24 (1)
Scheduled backward class	0 (0)	4 (<1)	4 (<1)
Other	372 (38)	357 (35)	729 (36)
Born in Mumbai	706 (73)	732 (71)	1438 (72)
Schooling			
Out of school	109 (11)	182 (18)	291 (15)
In school	860 (88)	845 (82)	1705 (85)
Never started school	4 (<1)	5 (<1)	9 (<1)
Does paid work	101 (10)	43 (4)	144 (7)
Part-time	51 (50)	28 (65)	79 (55)
Full-time	48 (48)	15 (35)	63 (44)
Missing	2 (2)	0 (0)	2 (1)
Works every day	44 (44)	28 (65)	72 (50)
Garment manufacture	10 (10)	2 (5)	12 (8)
Embroidery	8 (8)	3 (7)	11 (8)
Office assistant	9 (9)	1 (2)	10 (7)
Piecework	8 (8)	1 (2)	9 (6)
Daily income			
<Rs 100 (~US$1.5)	15 (15)	18 (42)	33 (23)
Rs 100–200 (~US$1.5–3.0)	44 (44)	14 (33)	58 (40)
>Rs 200 (~US$3.0)	30 (30)	7 (16)	37 (26)
Variable	5 (5)	2 (5)	7 (5)
Use internet at least once a week	380 (39)	164 (16)	544 (27)
Home internet	106 (11)	80 (8)	186 (9)
Mobile internet	379 (39)	94 (9)	473 (24)
Internet café	100 (10)	83 (8)	183 (9)
School internet	13 (1)	36 (3)	49 (2)
Respondents	973 (100)	1032 (100)	2005 (100)

### What do adolescents think of their rights?

We asked adolescents about their awareness of their rights to education, protection, participation, free speech, and play. If they had heard of a right, we asked them whether it applied more to boys or girls (Table 2). Although awareness – or understanding of the idea of rights – was higher in the older age group, greater proportions of girls than of boys said that they were aware of their rights at all ages. Levels of awareness were, nevertheless, not high. Only 63% of older girls were familiar with the right to education and 59% with the right to free speech. Of those who said that they had heard of individual rights, very few said that they applied differentially across the sexes. In situations of household food insecurity, most girls (90%) said that sons and daughters should have equal access to what there was to eat. Where there were differences in opinion between the sexes, boys were more likely than girls to suggest that daughters should have priority access to food (95% confidence interval for difference 15–23%). This pattern was not repeated when adolescents were asked about who should share in limited household cash. Again, girls were more likely to say that money should be shared equally between brothers and sisters, but boys who opted for one or the other were roughly equally divided between brothers and sisters as priority recipients. A greater proportion of girls said that they should be restricted in their choice of clothing (36%; 95% CI 24–47%).

**Table 2.** Adolescents’ familiarity with human rights and opinions on gendered allocations within the family.

	Male 11–14 y (%)	Female 11–14 y (%)	Male 15–19 y (%)	Female 15–19 y (%)	11–14 y (%)	15–19 y (%)	Difference % (95% CI)	Male (%)	Female (%)	Difference (95% CI)	All (%)
Aware of right to:											
Education	109 (24)	250 (54)	258 (50)	358 (63)	359 (39)	616 (57)	18 (13, 22)	367 (38)	608 (59)	21 (15, 27)	975 (49)
Protection	87 (19)	214 (46)	211 (41)	307 (54)	301 (33)	518 (48)	15 (11, 19)	298 (31)	521 (50)	20 (14, 26)	819 (41)
Participation	65 (14)	205 (44)	177 (34)	300 (53)	270 (29)	477 (44)	15 (11, 18)	242 (25)	505 (49)	24 (19, 29)	747 (37)
Free speech	64 (14)	221 (47)	178 (34)	332 (59)	285 (31)	510 (47)	16 (12, 20)	242 (25)	553 (54)	29 (24, 34)	795 (40)
Play	101 (22)	221 (47)	227 (44)	309 (55)	322 (35)	536 (49)	14 (10, 19)	328 (34)	530 (51)	18 (12, 23)	858 (43)
If food is limited at home, who should get it?											
Son	24 (5)	14 (3)	16 (3)	15 (3)	38 (4)	31 (3)	−1 (–3, 0)	40 (4)	29 (3)	−1 (–3, 1)	69 (3)
Daughter	116 (25)	34 (7)	135 (26)	35 (6)	150 (16)	170 (16)	−1 (–4, 2)	251 (26)	69 (7)	−19 (–23, −15)	320 (16)
Equal share	314 (69)	417 (89)	364 (70)	516 (91)	731 (80)	880 (81)	2 (–2, 6)	678 (70)	933 (90)	20 (16, 25)	1611 (81)
Don’t know	1 (<1)	1 (<1)	3 (<1)	0 (0)	2 (<1)	3 (<1)		4 (<1)	1 (<1)		5 (<1)
If money for education is limited at home, who should get it?											
Son	74 (16)	17 (4)	82 (16)	26 (5)	91 (10)	108 (10)	0 (–2, 2)	156 (16)	43 (4)	−12 (–15, −8)	199 (10)
Daughter	75 (16)	41 (9)	88 (17)	46 (8)	116 (13)	134 (12)	0 (–3, 3)	163 (17)	87 (8)	−8 (–11, −5)	250 (12)
Equal Share	305 (67)	407 (87)	344 (66)	492 (87)	712 (77)	836 (77)	0 (–3, 4)	649 (67)	899 (87)	20 (15, 25)	1548 (77)
Don’t know	1 (<1)	1 (<1)	4 (<1)	2 (0)	2 (<1)	6 (<1)		5 (<1)	3 (<1)		8 (<1)
Should there be a dress code for girls?											
Yes	191 (42)	362 (78)	221 (43)	441 (78)	553 (60)	662 (61)	1 (–2, 5)	412 (42)	803 (78)	36 (24, 47)	1215 (61)
Don’t know	4 (<1)	4 (<1)	9 (<1)	4 (0)	8 (<1)	13 (1)		13 (1)	8 (<1)		21 (1)
Respondents	455 (100)	466 (100)	518 (100)	566 (100)	921 (100)	1084 (100)		973 (100)	1032 (100)		2005 (100)

### Friendship and sex in adolescence

When we asked adolescents about their opinions on friendship between the sexes, 59% of males thought it was acceptable, compared with 36% of girls (95% CI for difference 18–31%: Table 3). Just how much our respondents knew about sex was elusive. While it was predictable that younger boys and girls would not be able to say what sex was, and while 84% of older boys said that they did, only 38% of older girls said so. Under half of adolescents said that they knew what was meant by the word ‘sex’ (828: 41%). Males were more likely to say so (552/973: 57% overall and 433/518: 84% of older males) than females (276/1032: 27% overall and 214/566: 38% of older females). Among multiple articulations, the commonest were physical interaction, sexual intercourse, inserting an organ in a private part (32%), getting close in a relationship (15%), kissing and touching (15%), and reproduction (8%). The difference between sex and love was often blurred: some young people described sex with expressions such as ‘girl and boy doing in bed sheets’ or ‘feeling hot’, while others used expressions such as ‘give and take happiness’ and ‘love story’.

**Table 3.** Adolescents’ opinions on sexuality, attraction, and sexual behavior.

	Male 11–14 y (%)	Female 11–14 y (%)	Male 15–19 y (%)	Female 15–19 y (%)	11–14 y (%)	15–19 y (%)	Difference % (95% CI)	Male (%)	Female (%)	Difference % (95% CI)	All (%)
It is ok for girls or boys to have friends of the opposite sex?											
	252 (55)	157 (34)	325 (63)	218 (39)	409 (44)	543 (50)	5 (1, 10)	577 (59)	375 (36)	−25 (–31, −18)	952 (47)
It is ok for boys to have multiple sexual partners											
	13 (3)	9 (2)	35 (7)	25 (4)	22 (2)	60 (6)	3 (1, 5)	48 (5)	34 (3)	−2 (–4, 0)	82 (4)
It is ok for girls to have multiple sexual partners											
	9 (2)	9 (2)	27 (5)	23 (4)	18 (2)	50 (5)	3 (1, 4)	36 (4)	32 (3)	−1 (–3, 1)	68 (3)
Sexual activity in adolescence is ok											
Yes	4 (1)	2 (<1)	36 (7)	5 (1)	6 (<1)	41 (4)	3 (2, 5)	40 (4)	7 (<1)	−4 (–5, −2)	47 (2)
It depends	15 (3)	4 (<1)	23 (4)	11 (2)	19 (2)	34 (3)	1 (0, 2)	38 (4)	15 (1)	−3 (–4, −1)	53 (3)
Missing	55 (12)	30 (8)	20 (4)	24 (4)	94 (10)	44 (4)		75 (4)	63 (6)		138 (7)
Know someone who is sexually active											
	33 (7)	19 (4)	115 (22)	43 (8)	52 (6)	158 (15)	9 (6, 12)	148 (15)	62 (6)	−9 (–12, −7)	210 (10)
If attracted to a person of the opposite sex, would:											
Do nothing	241 (53)	321 (69)	120 (23)	282 (50)	562 (61)	402 (37)	−24 (–28, −20)	361 (37)	603 (58)	21 (16, 26)	964 (48)
Attempt contact	82 (18)	61 (13)	192 (37)	107 (19)	143 (16)	299 (28)	12 (8, 16)	274 (28)	168 (16)	−12 (–17, −7)	442 (22)
Be friends	65 (14)	42 (9)	131 (25)	100 (18)	107 (12)	231 (21)	10 (6, 13)	196 (20)	142 (14)	−6 (–11, −2)	338 (17)
Ask them out	59 (13)	33 (7)	137 (26)	66 (12)	92 (10)	203 (19)	9 (6, 11)	196 (20)	99 (10)	−10 (–15, −6)	295 (15)
See if they are attracted	28 (6)	2 (<1)	84 (16)	4 (<1)	30 (3)	88 (8)	5 (3, 7)	112 (12)	6 (<1)	−11 (–15, −7)	118 (6)
Stare at them	8 (2)	0 (0)	42 (8)	1 (<0)	8 (<1)	43 (4)	3 (2, 4)	50 (5)	1 (<1)	−5 (–7, −3)	51 (3)
Follow them	11 (2)	0 (0)	32 (6)	0 (0)	11 (1)	32 (3)	2 (0, 3)	43 (4)	0 (0)	−4 (–6, −3)	43 (2)
Have heard about men or women touching themselves											
	102 (22)	17 (4)	387 (75)	63 (11)	119 (13)	450 (42)	29 (25, 32)	489 (50)	80 (8)	−43 (–47, −39)	569 (28)
Think this is healthy	20 (4)	2 (<1)	82 (16)	9 (2)	22 (2)	91 (8)	6 (4, 9)	102 (10)	11 (1)	−10 (–13, −7)	113 (6)
Are in a relationship	32 (7)	6 (1)	119 (23)	25 (4)	38 (4)	144 (13)	9 (7, 11)	151 (15)	31 (3)	−13 (–15, −11)	182 (9)
Parents know and approve of the relationship											
	9 (2)	1 (<1)	22 (4)	8 (1)	10 (1)	30 (3)	3 (0, 6)	31 (3)	9 (1)	−4 (–7, −2)	40 (2)
Have heard of men who have sex with men or women who have sex with women											
	87 (19)	156 (33)	345 (67)	313 (55)	243 (26)	658 (61)	34 (31, 38)	432 (44)	469 (45)	1 (–5, 7)	901 (45)
Have heard of bisexual people											
	62 (14)	103 (22)	291 (56)	228 (40)	165 (18)	519 (48)	30 (26, 33)	353 (36)	331 (32)	−4 (–10, 1)	684 (34)
Have heard of heterosexual people											
	335 (74)	422 (91)	483 (93)	559 (99)	757 (82)	1042 (96)	14 (11, 17)	818 (84)	981 (95)	11 (6, 16)	1799 (90)
Consider men who have sex with men or women who have sex with women normal											
	11 (2)	3 (<1)	21 (4)	36 (6)	14 (2)	57 (5)	4 (2, 5)	32 (3)	39 (4)	0 (–2, 3)	71 (4)
Consider bisexual people normal											
	15 (3)	3 (1)	31 (6)	26 (5)	18 (2)	57 (5)	3 (2, 5)	46 (5)	29 (3)	−2 (–4, 0)	75 (4)
Consider heterosexual people normal											
	308 (68)	391 (84)	452 (87)	493 (87)	699 (76)	945 (87)	11 (8, 14)	760 (78)	884 (86)	8 (2, 13)	1644 (82)
Respondents	455 (100)	466 (100)	518 (100)	566 (100)	921 (100)	1084 (100)		973 (100)	1032 (100)		2005 (100)

Adolescents of both sexes were almost unanimous in saying that it was unacceptable for either boys or girls to have multiple sexual partners. Over 90% said that premarital sex was unacceptable, and almost all said that their parents would not condone it. 88% said that sexual activity was unacceptable in adolescence. Few (10%) said that they knew someone who was sexually active: 15% of older males and 6% of older females (95% CI for difference 7–12%). If they were attracted to someone, the largest group (48%) would do nothing (37% of boys and 58% of girls; difference 16–26%), 22% would try to make contact in some way, and 15% would ask them out (20% of boys and 10% of girls; difference 6–15%). 75% of older boys were familiar with the idea of masturbation (although 79% said that it was unhealthy), but only 11% of older girls said that they were.

Thirteen percent of older respondents said that they were in a relationship (23% of older males and 4% of older females). Twelve percent more males than females disclosed this (difference 11–15%). Six percent of older girls and 11% of older boys said that their parents would allow them to go out with someone, and 3% of older adolescents said that their parents knew about their partners. Under half of respondents said that they had heard of men or women who preferred sex with the same gender, or bisexual people, 82% considered heterosexuality normal and over 95% said that other sexualities were abnormal.

### Sexual refusal and coercion

Pestering someone for a date was generally not considered acceptable, although 13% of older males thought it was (Table 4) and a greater proportion of males thought so at all ages (difference 4–10%). Few adolescents said that they felt pressure to be sexually active, but 6% of older boys and 2% of older girls said that they or someone they knew had been coerced at some point. Nonetheless, only 78% of older adolescents felt that it was reasonable to refuse to have sex 79% saying that coercion would be a form of violence. Teasing a person of a different sex was unacceptable (97%). A greater proportion of girls (85%) than boys (61%) thought that when a girl said no to sex she meant it (difference 19–30%). Around two-fifths of boys felt that this was not the case or that it depended on the circumstances.

**Table 4.** Adolescents’ opinions on sexual refusal and coercion.

	Male 11–14 y (%)	Female 11–14 y (%)	Male 15–19 y (%)	Female 15–19 y (%)	11–14 y (%)	15–19 y (%)	Difference % (95% CI)	Male (%)	Female (%)	Difference % (95% CI)	All (%)
Pestering someone for a date is acceptable											
	26 (6)	8 (2)	66 (13)	22 (4)	34 (4)	88 (8)	4 (2,6)	92 (9)	30 (3)	−7 (–10, −4)	122 (6)
Feel pressure to be sexually active	6 (1)	1 (<1)	20 (4)	6 (<1)	7 (<1)	26 (2)	2 (1, 3)	26 (3)	7 (<1)	−2 (–3, −1)	33 (2)
Have ever felt coerced into sex or know someone who has been											
	13 (3)	10 (2)	31 (6)	13 (2)	23 (3)	44 (4)	1 (0, 3)	44 (5)	23 (2)	−2 (–4, −1)	67 (3)
It is ok to say no in such a situation											
Yes	290 (64)	357 (77)	407 (79)	443 (78)	647 (70)	850 (78)	8 (5, 11)	697 (72)	800 (78)	5 (–2, 13)	1497 (57)
Don’t know	103 (23)	27 (6)	37 (7)	12 (2)	130 (14)	49 (5)		140 (14)	39 (4)		179 (9)
Missing	19 (4)	14 (3)	16 (3)	15 (3)	33 (4)	31 (3)		35 (4)	29 (3)		64 (3)
If one partner does not want to have sex, if one person still does it, it is still violence											
Yes	300 (66)	331 (71)	410 (79)	449 (79)	631 (69)	859 (79)	10 (7, 14)	710 (73)	780 (76)	2 (–4, 8)	1490 (74)
Sometimes	3 (<1)	1 (<1)	0 (0)	0 (0)	4 (<1)	0 (0)	0 (–1, 0)	3 (<1)	1 (<1)	0 (–1, 0)	4 (<1)
Don’t know	78 (17)	50 (11)	18 (3)	18 (3)	128 (14)	34 (3)		94 (10)	68 (7)		162 (8)
Missing	20 (4)	13 (3)	16 (3)	16 (3)	33 (4)	31 (3)		35 (4)	29 (3)		64 (3)
Teasing members of the opposite or same sex is ok											
	10 (2)	13 (3)	16 (3)	14 (2)	23 (3)	30 (3)	0 (–1, 2)	26 (3)	27 (3)	0 (–2, 2)	53 (3)
When a girl says no to a boy, she means no											
Yes	281 (62)	385 (83)	310 (60)	493 (87)	666 (72)	803 (74)	1 (–2, 5)	591 (61)	878 (85)	24 (19, 30)	1469 (73)
Depends	60 (13)	13 (3)	107 (21)	17 (3)	73 (8)	124 (11)	3 (1, 6)	167 (17)	30 (3)	−14 (–17, −11)	197 (10)
Missing	6 (1)	7 (1)	5 (<1)	3 (<1)	13 (1)	8 (<1)		11 (1)	10 (<1)		21 (1)
Respondents	455 (100)	466 (100)	518 (100)	566 (100)	921 (100)	1084 (100)		973 (100)	1032 (100)		2005 (100)

### Communication with parents

Most adolescents said that their parents allowed them to choose their own friends, although males were more likely to say this (89%) than females (75%; difference 10–19%. Table 5). Around two-thirds were allowed to go out with their friends, but friends of another sex were less permissible (42% of males and 17% of females; difference 20–29%)) and meeting them without others present less so (11%). More than 85% of respondents said that they had discussed their studies with their parents, but more females (38%) than males (15%) said that they found this difficult (difference 15–31%). Most (79%) said that they had discussed their career, irrespective of gender, although, again, females (27%) tended to find this difficult (difference 2–11%). Females were more likely (12%) than males (7%) to say that they had discussed marriage (difference 1–8%). Males particularly said that they found it difficult to discuss their sexuality (88%).

**Table 5.** Adolescents’ opinions on communication with their parents.

	Male 11–14 y (%)	Female 11–14 y (%)	Male 15–19 y (%)	Female 15–19 y (%)	11–14 y (%)	15–19 y (%)	Difference % (95% CI)	Male (%)	Female (%)	All (%)
Parents allow choice of friends	396 (87)	350 (75)	472 (91)	422 (75)	746 (81)	894 (82)	1 (–2, 4)	868 (89)	772 (75)	1640 (82)
Parents allow adolescent to go out with friends	265 (58)	299 (64)	396 (76)	390 (69)	564 (61)	786 (73)	11 (6, 16)	661 (68)	689 (67)	1350 (67)
Parents allow friends of opposite sex	146 (32)	74 (16)	260 (50)	106 (19)	220 (24)	366 (34)	10 (5, 14)	406 (42)	180 (17)	586 (29)
Parents allow meeting friends of opposite sex alone	14 (3)	30 (6)	78 (15)	33 (6)	44 (5)	111 (10)	5 (3, 7)	92 (9)	63 (6)	204 (11)
Parents allow boyfriend or girlfriend	20 (4)	22 (5)	55 (11)	34 (6)	42 (5)	89 (8)	4 (2, 5)	75 (8)	56 (5)	131 (6)
Have discussed future studies with parents	387 (85)	426 (91)	441 (85)	477 (85)	813 (88)	918 (85)	−3 (–7, −1)	828 (85)	903 (87)	1731 (87)
Have discussed future career with parents	314 (69)	393 (84)	412 (79)	453 (80)	707 (77)	865 (80)	3 (–1, 6)	726 (75)	846 (82)	1572 (79)
Have discussed marriage with parents	24 (5)	17 (4)	46 (9)	103 (18)	41 (4)	149 (14)	9 (7, 12)	70 (7)	120 (12)	190 (10)
Find it difficult to discuss studies	69 (15)	176 (38)	77 (15)	219 (39)	245 (27)	296 (27)	1 (–3, 4)	146 (15)	395 (38)	541 (27)
Find it difficult to discuss career	101 (22)	122 (26)	93 (18)	153 (27)	223 (24)	246 (23)	−2 (–5, 2)	194 (20)	275 (27)	469 (24)
Find it difficult to discuss sexuality	398 (87)	313 (67)	455 (88)	406 (72)	711 (77)	861 (79)	2 (–1, 6)	853 (88)	719 (70)	1572 (78)
Find it difficult to discuss life	142 (31)	117 (25)	126 (24)	152 (27)	259 (28)	278 (26)	−2 (–6, 1)	268 (28)	269 (26)	537 (27)
Feel able to discuss sexuality	32 (7)	74 (16)	85 (16)	118 (21)	106 (12)	203 (19)	7 (4, 10)	117 (12)	192 (19)	309 (15)
Best form of marriage										
Arranged marriage	320 (70)	339 (73)	304 (59)	385 (68)	659 (72)	689 (64)	−8 (–12, −4)	624 (64)	724 (70)	1348 (67)
Love marriage	39 (9)	16 (3)	82 (16)	30 (5)	55 (6)	112 (10)	4 (2, 7)	121 (12)	46 (4)	167 (8)
Arranged love marriage	21 (5)	8 (2)	38 (7)	39 (7)	29 (3)	77 (7)	4 (2, 6)	59 (6)	47 (5)	106 (5)
Undecided	75 (16)	103 (22)	94 (18)	112 (20)	178 (19)	206 (19)		169 (17)	215 (21)	384 (19)
Parents’ idea of best form of marriage										
Arranged marriage	406 (89)	437 (94)	456 (88)	525 (93)	843 (92)	981 (90)	−1 (–3, 1)	862 (89)	962 (93)	1824 (91)
Love marriage	23 (5)	14 (3)	26 (5)	10 (2)	37 (4)	36 (3)	−1 (–2, 1)	49 (5)	24 (2)	73 (4)
Arranged love marriage	14 (3)	11 (2)	24 (5)	28 (5)	25 (3)	52 (5)	2 (1, 3)	38 (4)	39 (4)	77 (4)
Undecided	12 (3)		12 (2)	3 (<1)	16 (2)	15 (2)		24 (2)	7 (<1)	31 (1)
Parents would let them marry person of their choice	50 (11)	15 (3)	132 (25)	33 (6)	65 (7)	165 (15)	8 (6, 10)	182 (19)	48 (5)	230 (11)
Right age for men to marry										
15–17 y	4 (<1)	1 (<1)	0 (0)	1 (<1)	5 (<1)	1 (<1)	0 (–1, 0)	4 (<1)	2 (<1)	6 (<1)
18–21 y	124 (27)	56 (12)	127 (25)	45 (8)	180 (20)	172 (16)	−4 (–8, 3)	251 (26)	101 (10)	352 (18)
22–25 y	250 (55)	233 (50)	305 (59)	287 (51)	483 (52)	592 (55)	2 (–4, 7)	555 (57)	520 (50)	1075 (54)
Over 25 y	63 (14)	142 (30)	80 (15)	214 (38)	205 (22)	294 (27)	5 (1, 8)	143 (15)	356 (34)	499 (25)
Undecided	14 (3)	34 (7)	6 (1)	19 (2)	48 (6)	25 (2)	0 (–1, 1)	20 (2)	53 (6)	73 (2)
Right age for women to marry										
15–17 y	11 (2)	10 (2)	5 (<1)	2 (<1)	21 (2)	7 (<1)	−2 (–3, −1)	16 (2)	12 (1)	28 (1)
18–21 y	286 (63)	276 (59)	367 (71)	335 (59)	562 (61)	702 (65)	3 (–1, 7)	653 (67)	611 (59)	1264 (63)
22–25 y	113 (25)	130 (28)	115 (22)	189 (33)	243 (26)	304 (28)	1 (–3, 6)	228 (23)	319 (31)	547 (27)
Over 25 y	25 (5)	22 (5)	24 (5)	31 (5)	47 (5)	55 (5)	0 (–2, 1)	49 (5)	53 (5)	102 (5)
Undecided	20 (5)	28 (6)	7 (2)	9 (3)	48 (6)	16 (2)		27 (3)	53 (4)	64 (4)
Respondents	455 (100)	466 (100)	518 (100)	566 (100)	921 (100)	1084 (100)		973 (100)	1032 (100)	2005 (100)

**Figure 1.**
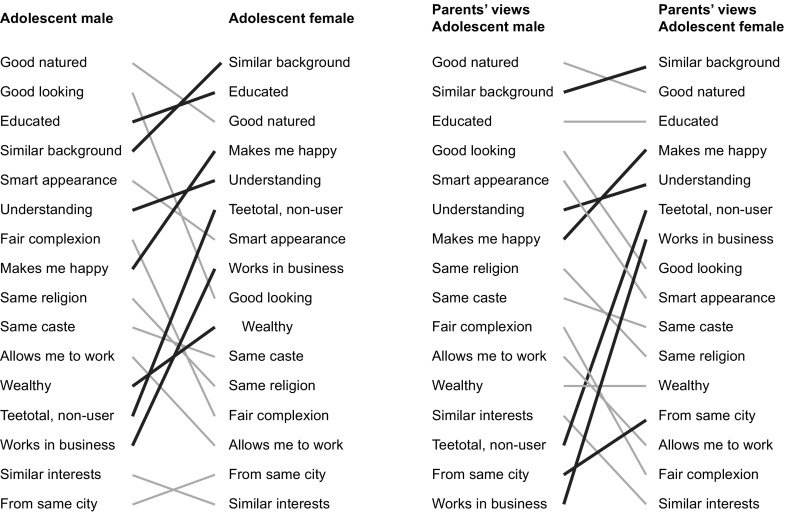
Adolescents’ expectations of future life partners, and opinions on their parents’ expectations, in rank order.

Adolescents said that the best form of marriage was arranged (67%) and that their parents thought so too (91%). Nineteen percent of males said that their parents would allow them to marry a partner of their choice, but this opinion was shared by only 5% of females (difference 11–18%). Adolescents’ expectations of their future life partners differed in some respects (Figure 1). Education, pleasant nature, and an understanding character were important to both sexes. Females were more likely to prioritise family background, men who made them happy, non-drinkers and non-users of drugs, work in business, and wealth. Males were more likely to prioritise looks, smart appearance, fair complexion, and religion. That men should not be drinkers or drug-users was much more important to women. Most of these requirements were echoed in respondents’ ideas about what their parents would prefer. Family background was important, and females gave more priority to residence in the same city.

## Discussion

### Summary of findings

Our interviews with 2005 adolescents provide a snapshot of urban transitional India, in which aspirational families in informal settlements often send their children to private English medium schools (sons more than daughters) and underage labour is becoming less common. Young people have a limited understanding of their rights - although in this they may be no different from young people in other countries – and take at least superficially gender-equitable views of household food and finance allocation.

### Norms

If we take their answers at face value, young women articulated a correspondence between injunctive norms, normative expectations, and empirical norms. They stayed away from young men, knew little about sex and didn’t masturbate, disapproved of premarital relationships and multiple partners, accepted gendered dress codes (also found in the ARSH study) (Nair, Leena, George, Thankachi, & Russell, 2013a), did not discuss their future career much with their parents, and expected an arranged marriage with a man approved of by their parents, perhaps from the same city so that they could see their parents easily. This man would be from a compatible family background, educated and employed, and (hopefully) not a drinker. Their views represented, perhaps, limited manoeuvring on a background of an assumed gender role in which family control was prominent (Fatusi & Hindin, 2010; Jejeebhoy, 1998).

Young men were more likely to approve of friendships with women, say that they knew something about sex (a trait described elsewhere) (Fatusi & Hindin, 2010; WHO, 2011), be amenable to asking women out, be likely to be in a relationship, and be less convinced that no meant no. They were, however, uncomfortable with the idea of masturbation and also disapproved of premarital relationships and multiple partners, as has been found in other studies (Guilamo-Ramos et al., 2012). The Hindi word for masturbation, *swapnadosh*, means ‘fault of dreams’, and a sense of shame around it may reflect a culture in which sexuality and pleasure remain largely unexplored (Nair et al., 2013a).

These were not, to put it mildly, rebellious teenagers. Braggadocio was not the hallmark of their answers to our questions about their relationships and sexuality. What interested us was their apparent naivety and wholesomeness, manifest in a romanticism that characterized descriptions of sex. The culture of family solidarity, obeisance to parents, suppression of career priorities to the role as homemaker and mother, and appearance as a good woman appeared robust to the climate of sexual harassment, pornography, and television in which young people lived. Opinions about sexual activity were hetero-normative and conformed with societal norms, supporting abstinence until marriage and disapproving of masturbation, if they knew about it. There was some cognitive dissonance: coercion was frowned upon and largely unreported by young women, while young men said that it was unreasonable to refuse sex.

The young people we interviewed all lived in an informal settlement. We do not need to expand here on the difference between their lived reality – its emphasis on education and bourgeois and family aspirations – and the salaciously orientalised views of informal settlements as nexuses of transgression (criminal and sexual) that have characterized societies at least since Victorian London and New York. It is, however, interesting to speculate that precisely because of their families’ lack of wherewithal these young men and women might have had more traditional views and aspirations than their counterparts among the wealthy.

### Concerns

We have some concerns about our data-set that raise a number of provocative questions. First, asking young people about sex when they have been raised in an environment in which empirical norms are overlooked and injunctive norms constantly reiterated may lead to best behaviour bias. Young people’s responses may have been processed at three levels: what they really believed, what they thought their parents believed, and what they thought that wider society expected.

Although the interviews were conducted by people of the same sex (McCombie & Anarfi, 2002), face-to-face interviews present their own set of challenges, even if culturally appropriate (Jejeebhoy, 1998). Interviewers were from the same community as respondents, and were trained and supervised carefully, but it is possible that their own attitudes could have cued certain responses. We note, however, that the urge to give politically correct answers did not extend to young people saying that they were comfortable with the idea of non-heterosexual relationships. This raises the possibility that cultural norms might have overridden contemporary ones. We were at first critical of our findings, but over months of re-examination and the implementation of our intervention to engage with adolescents, we now wonder whether their answers were honest and their relative lack of sexual knowledge and activity genuine.

When we designed the study, we were aware that inclusion of adolescents with disabilities was important. We trained the interviewers to identify and ask questions to adolescents with locomotor, visual, hearing, cognitive, and learning difficulties. Unfortunately, time pressure and lack of confidence on the part of interviewers meant that these groups were not represented adequately. Given our interest in inclusion, this was a lesson. We have prioritised inclusion in our subsequent work. Interviewers will follow protocols to identify disability, we will check the numbers regularly, and we will employ experts in research with people with disabilities to help with the interviews.

### Final thoughts

Probably our biggest lesson from the study and our subsequent work is that research involves allaying the fears of a number of stakeholders beyond the participants. In developing the questionnaire, we were careful to respond to the concerns of colleagues and institutional review boards that direct questions about sexuality might be inappropriate. Adolescent gender and sexuality are relatively new areas for work in India, added to which are (we think, largely unfounded) concerns about the sensitivities of slum-dwelling communities. Parents whom we consulted in developing the study and before and during our program were generally enthusiastic about them, and it may be that the obliquity of some of the questions – for example, asking adolescents if they knew others who were in sexual relationships – would have been best avoided. It is likely that over a decade of engagement with the community has contributed to uptake of program activities, but it has been our experience that respondents welcome the opportunity to discuss the issues and are keen to be involved in subsequent activities. We have found that questions about adolescents’ own experiences, asked in a supportive atmosphere, often yield direct answers. We are reasonably convinced that they are served better by respectful directness, and are acting on this in our current work.

## Disclosure statement

None of the authors has a conflict of interest.

## Funding

This work was supported by the Ford Foundation [grant number 0135-0632]. David Osrin was supported by the Wellcome Trust [grant number 091561/Z/10/Z]. The funders played no part in study design, collection, analysis or interpretation of data, in the writing of the report, or in the decision to submit the article for publication. Nayreen Daruwalla had full access to the study data and final responsibility for the decision to submit for publication.

## Notes on contributors

***Nayreen Daruwalla*** is Director of the Program on Prevention of Violence Against Women and Children at SNEHA. She led the development of the adolescent program and works on gender, sexuality, health, and wellbeing.

***Tanvi Mishra*** was a research officer at SNEHA. She managed the survey described in the article. She is interested in gender and sexuality as subjects for research and journalism.

***Neeta Karandikar*** is Associate Program Director for Empowerment, Health and Sexuality of Adolescents at SNEHA (EHSAS). She is interested in adolescent health.

***Shanti Pantvaidya*** is Executive Director, Programs at SNEHA. She is interested in adolescent health and integration of community and health service activities.

***David Osrin*** is Professor of Global Health at University College London. He studies community action, gender-based violence, and urban health.
